# Do we need new trials of procalcitonin-guided antibiotic therapy?

**DOI:** 10.1186/s13054-018-1948-6

**Published:** 2018-01-27

**Authors:** Thiago Lisboa, Jorge Salluh, Pedro Povoa

**Affiliations:** 1 Rede Institucional de Pesquisa e Inovação em Medicina Intensiva, Complexo Hospitalar Santa Casa de Misericordia de Porto Alegre, Porto Alegre, Brazil; 2 Critical Care Department and Infection Control Committee, Hospital de Clinicas de Porto Alegre, Porto Alegre, Brazil; 3 Instituto D’OR de pesquisa e ensino, Rio de Janeiro, Brazil; 4 Programa de pós-graduação de Clinica Medica Universidade Federal do Rio de Janeiro—UFRJ, Rio de Janeiro, Brazil; 5 Unidade de Cuidados Intensivos Polivalente, Hospital de São Francisco Xavier, Centro Hospitalar de Lisboa Ocidental, Lisboa, Portugal; 6 NOVA Medical School, CEDOC, Universidade Nova de Lisboa, Lisboa, Portugal

## Abstract

Using biomarkers as a guide to tailor the duration of antibiotic treatment in respiratory infections is an attractive hypothesis assessed in several studies. Recent work aiming to summarize the evidence assessed the effect of a procalcitonin (PCT)-guided antibiotic treatment on outcomes in acute lower respiratory tract infections (LRTI), suggesting that significant reductions in antibiotic duration occur when using a PCT-guided algorithm. However, controversial evidence also suggested PCT-guided algorithms were associated with increased antibiotic duration and increased incidence of *Clostridium difficile*, without any impact on mortality, in real-world settings. So, although using PCT-guided antibiotic stewardship is promising, after more than a decade of randomized controlled trials on this topic the evidence in its favor is still less than compelling due to limitations in trial design, not taking into consideration fundamental aspects of PCT biology, and the absence of evidence-based antimicrobial duration in intervention and control groups. In this commentary we highlight some questions and limitations of primary PCT study data that might impact interpretation and clinical use of PCT at the bedside.

## Introduction

Using biomarkers as a guide to tailor the duration of antibiotic treatment in respiratory infections is an attractive hypothesis assessed in several studies. A recent meta-analysis aiming to summarize the evidence assessed the effect of a procalcitonin (PCT)-guided antibiotic treatment on outcomes in acute lower respiratory tract infections (LRTI) suggested significant reductions in antibiotic duration occur when using a PCT-guided algorithm [1]. However, its use in “real-world” conditions was recently challenged by Chu et al. [2], who found that the use of PCT-guided algorithms was associated with increased antibiotic duration and increased incidence of *Clostridium difficile*, without any impact on mortality, in real-world settings in the US. In this commentary, we highlight some questions and limitations of primary PCT study data that might impact interpretation and clinical use of PCT at the bedside.

## Are control groups receiving the best care?

A major concern in PCT-guided trials is antibiotic use in the control group. According to the World Medical Association’s Helsinki declaration, “the benefits, risks, burdens, and effectiveness of a new intervention must be tested against those of the best current proven intervention,” but defining “best current proven intervention” is difficult [3]. Heterogeneity of current practices has been a major argument against using routine care without any constraints as the comparator. When we consider duration of antibiotic treatment and/or antibiotic exposure, the duration of therapy in control groups is systematically above those recommended by guidelines and the best available evidence base (e.g., standard of care for ventilator-associated pneumonia (VAP) patients in the control group should be 6–8 days, not 13 days [4, 5]). Should any intervention compared to a “suboptimal” standard of care (even when it is usual care) be recommended/adopted in clinical practice?

For instance, usual care is sometimes far from the best available care or what should be standard of care. Trials using a protocolized rather than an unrestricted standard care control group will likely have enhanced validity as long as the protocolized care control group is representative of standard care practices [6]. In PCT studies, a protocolized group with clear stop rules for antibiotic duration, making it more compatible with best available evidence and recommendations, would be important and could lead to better evaluation of biomarker-based antimicrobial treatment [7].

## Are PCT algorithms really followed?

Also, PCT algorithms consider that a PCT < 0.1 μg/L bacterial infection is very unlikely and antibiotics should not be prescribed or should be withheld. It is well known that in several bacterial infections, for example, VAP [8, 9], PCT is not a good marker of diagnosis since it presents a high rate of false negatives. However, it was with some surprise that we realized that the rate of PCT false negatives among the patients diagnosed with LRTI included in the 32 randomized controlled trials (RCT) of the above-mentioned meta-analysis was well above 30% [1]! The authors did not give this information according to the setting nor according to the infection. In the ICU setting, doctors almost always wisely overrule this recommendation, as was very clear in the PRORATA trial (overruling the algorithm at inclusion 21% of the time) [10], since the “blind” application of the algorithm could be unsafe. Also, very low PCT levels on enrolment (>40% PCT < 0.25 μg/L) raises another problem. Every PCT algorithm is based on the assessment of absolute and/or relative variations of PCT measurements during the course of antibiotic therapy in relation to the baseline value. This so-called lack of amplitude of variation of PCT is another limitation of its clinical applicability not discussed in the study.

## Are PCT limitations addressed?

In addition, another important limitation of PCT use is related to the lack of information on specific conditions and populations where its value is inadequate because of intrinsic constraints. In critically ill patients the presence of acute kidney injury or the use of renal replacement therapies has a profound effect on PCT concentrations [11–14]. Additionally, PCT tends to be less responsive to repeated inflammatory insults (such as VAP or nosocomial bloodstream infections), resulting in lower than expected peak concentrations [15]. Similar findings are described in neutropenic patients, limiting the interpretation and use of PCT in this context [16].

## Conclusions

Clinical use of biomarker-guided antibiotic stewardship such as PCT is promising, but after more than a decade of RCTs on the topic the evidence in its favor is still less than compelling due to limitations in trial design. These limitations include not taking into consideration fundamental aspects of PCT biology and the absence of evidence-based antimicrobial duration in intervention and control groups, namely in VAP. A double-trigger criteria, in which antibiotics are stopped according to the clinical course and either decreases in biomarker levels, according to an algorithm, or the completion of 5–7 days of treatment, whichever comes first [17], might be a safe and efficient strategy to decrease antimicrobial therapy duration in critically ill patients.
